# Renewable Polymeric Adhesives

**DOI:** 10.3390/polym9040126

**Published:** 2017-03-28

**Authors:** Antonio Pizzi

**Affiliations:** LERMAB, Laboratoire d’Etude et de Recherche sur le MAteriau Bois, Université de Lorraine, 27 rue Philippe Seguin, CS60036, 88021 Epinal, France; antonio.pizzi@univ-lorraine.fr

The field of renewable polymeric adhesives has become a very active field of research in the last few years. The cost increases in petrochemicals due to the foreseen future scarcity of oil has engendered a surge in the interest of using renewable materials in resins and adhesives. This trend encompasses all the different fields and applications of adhesives with a number of notable, different general approaches. From the more conventional approach of substituting part of some oil-derived synthetic materials in the adhesive with a renewable resource, approaches based on the preparation of adhesives by the total use of some renewable material without any synthetic oil-derived one have developed in a number of different directions and applications. To the simple preparation of adhesives by the usual synthesis but using chemicals that are produced from “biosourced” materials, are opposed even more extreme, self-adhesion approaches, which have also been proposed, studied, developed and applied in particular fields. It must be pointed out that, while there is a significant surge of research in this field, and an abundance of novel ideas, the translation of such ideas, formulations and approaches into industrial practice is still lower than for synthetic oil-derived adhesives. However, while this has been true for some years, a significant acceleration of renewable adhesives plant trials and industrial applications has occurred and is relentlessly increasing. In this context, it can be said that renewable adhesives are slowly but surely taking their place in a range of industrial adhesives. Both public awareness of environmental concerns, with the consequent hardening of legislative regulations, and real or medium-term foreseen increases in oil costs, have contributed to this surge.

This *Polymers* Special Issue presents a clear image of the variety of applications and approaches in this field. Several articles on lignin and protein adhesives for wood form part of the Special Issue; these articles are numerous simply because thermosetting wood adhesives constitute about 60%, by volume, of all adhesives used. In the same wood adhesives field, the use of sour cassava modified adhesives addresses the same concerns. In this same context and issue, the transformation of the most used wood panel adhesive in the world, namely urea-based aminoplastic adhesives, into an acceptable renewable adhesive—after all, urea is a renewable resource—by the use of ionic liquids as hardeners is a significant medium-term approach. In addition to the novel ideas for commodity type, bulk wood adhesives, even more revolutionary approaches have been presented in other fields, such as from mussel-imitating protein adhesives with various additives. Equally, the preparation of epoxy resins by the elimination of bisphenol-A using a non-toxic natural tannin material and the modification of epoxy resins by biochemical means is particularly notable in a field of great application interest. The use of chitosans and xanthan gum as binders of cork, the development of bio-based polyacetals developed from isosorbide, and lignin amine polyurethanes are no less revolutionary in their own fields. The partial substitution trend of a synthetic material is also very present in this Special Issue, with the partial substitution of biosourced furanic polymers for oil-derived phenolic ones. The issue also contains more theoretical studies on reaction mechanisms; after all, it is fine to talk about new adhesives, but it is also necessary to develop their theoretical base if they are to be more widely accepted. The trend of totally new cross-linking reactions in a renewable polymeric adhesive, providing a totally different approach, is also represented. Finally, a few reviews on important aspects are also presented, some of which are on revolutionary approaches, such as the work on tilted polymeric nanohairs adhesion—a biomimetics concept derived from the observation of how geckos are able to adhere to a vertical wall.

The topics presented in this Special Issue cover a wide range of new ideas, concepts, and approaches, and have been presented by real cutting edge specialists in this growing field. However, all these approaches are by no means exhaustive if one scans the world literature on renewable polymeric adhesives. All the above-mentioned approaches and many more can be found: from the older tannin-aldehyde adhesives—used in industrial applications in a few countries for several decades, and still in use—to newer cross-linking reactions and processes, to more revolutionary concepts such as wood welding without adhesives where the substrate itself functions as the adhesive under the particular conditions of application used.

To conclude, the explosion of interest, research, new ideas and new concepts in renewable adhesives is growing relentlessly but perhaps unevenly. While partial or total substitution of some synthetic adhesives by renewables will soon reach industrial application, in other cases industrial adoption may take more time or be more difficult. However, one thing is certain: the strong forward impulse to which this field is currently subject is now irreversible and these adhesives will continue to grow and occupy increasingly substantial parts of the market.

